# Environment-by-PGS Interaction in the Classical Twin Design: An Application to Childhood Anxiety and Negative Affect

**DOI:** 10.1080/00273171.2023.2228763

**Published:** 2023-07-13

**Authors:** Susanne Bruins, Jouke-Jan Hottenga, Michael C. Neale, René Pool, Dorret I. Boomsma, Conor V. Dolan

**Affiliations:** a Department of Biological Psychology, Vrije Universiteit; b Amsterdam Public Health research institute; c Virginia Institute for Psychiatric and Behavioral Genetics, Virginia Commonwealth University; d Amsterdam Reproduction and Development research institute

**Keywords:** Genotype-environment (g × e) interaction, moderation, mono- and dizygotic twins, polygenic scores (pgs), genetic covariance structure modeling

## Abstract

One type of genotype-environment interaction occurs when genetic effects on a phenotype are moderated by an environment; or when environmental effects on a phenotype are moderated by genes. Here we outline these types of genotype-environment interaction models, and propose a test of genotype-environment interaction based on the classical twin design, which includes observed genetic variables (polygenic scores: PGSs) that account for part of the genetic variance of the phenotype. We introduce environment-by-PGS interaction and the results of a simulation study to address statistical power and parameter recovery. Next, we apply the model to empirical data on anxiety and negative affect in children. The power to detect environment-by-PGS interaction depends on the heritability of the phenotype, and the strength of the PGS. The simulation results indicate that under realistic conditions of sample size, heritability and strength of the interaction, the environment-by-PGS model is a viable approach to detect genotype-environment interaction. In 7-year-old children, we defined two PGS based on the largest genetic association studies for 2 traits that are genetically correlated to childhood anxiety and negative affect, namely major depression (MDD) and intelligence (IQ). We find that common environmental influences on negative affect are amplified for children with a lower IQ-PGS.

## Introduction

Genotype-environment interaction occurs when the effects of environmental exposure on a trait systematically depend on an individual’s genotype, or when the effects of a genotype on a trait depend on an individual’s environmental exposure (Eaves 1977; Boomsma and Martin 2002). On the substantive level, genotype-environment interaction is relevant to our understanding of development across multiple domains, including psychopathology (Hicks et al. 2009; Samek et al. 2015, 2017; D. Molenaar et al. 2016), personality (Boomsma et al. 1999), or substance use (Koopmans et al. 1999; Dick et al. 2007). On the statistical level, genotype-environment interaction is relevant to the correct interpretation of parameters in the modeling of data from twins, families, or genotyped individuals. Unmodeled genotype-environment interaction, if present, may result in biased parameter estimates (Purcell 2002; Verhulst et al. 2019), that depend on the source of the interaction. Here we often distinguish between environmental factors that are common (C) to family members who share their environment, and environmental factors that are not shared between family members (E). An interaction of additive genotype (A) with common environmental factors (A × C interaction) presents as part of the additive genetic variance, and any A × E interaction presents as part of the unshared environmental variance.

The vast majority of genotype-environment interaction studies in behavioral science are conducted within a framework in which the effect of the genotype depends on a measured environmental exposure, as histotested by moderation models, where the environment moderates the influence of latent genetic and environmental factors on an outcome phenotype (Purcell 2002). For example, a religious upbringing diminishes genetic effects on disinhibition (Boomsma et al. 1999) as adolescent twins with a religious upbringing report lower levels of disinhibition, and genetic effects on disinhibition are smaller in this group. Genetic effects on the development of adolescent externalizing disorders appear to be amplified in the face of environmental stress (Hicks et al. 2009; Samek et al. 2017). This moderating effect of environmental stress is important in adolescence, but wanes in young adulthood (Johnson et al. 2009; Samek et al. 2015, 2017).

The study of G × E interaction in genetically informative designs has been facilitated by the application of structural equation modeling (SEM; Boomsma and Molenaar 1986; Molenaar and Boomsma 1987). Boomsma and Molenaar showed how the covariance between measures and between family members could be decomposed into genetic and environmental sources of variance and covariance using the LISREL software (Jöreskog 1973; Boomsma and Molenaar 1986), and D. Molenaar and Dolan (2014) illustrated how methods and approaches as developed in psychometrics proved to be inspirational in the study of G × E interaction.

Methods that investigate genotype-environment interaction as a function of a measured (environmental) moderator are relatively intuitive and straightforward to implement, as outlined by Purcell (2002). Peter Molenaar demonstrated that genotype-environment interaction in the classical twin design can also be detected by nonlinear factor analysis (Molenaar and Boomsma, 1987), and by analysis of the distribution of factor scores (Molenaar et al. 1990, 1999) in cross-sectional and longitudinal structural equation models, without the need to measure the environmental exposure (or the genotype). Inspired by Peter Molenaar’s work on factor scores, van der Sluis et al. (2012) and D. Molenaar et al. (2012a, 2013) proposed to study genotype-environment interaction by considering additive genetic factor (A) scores as moderators of environmental effects by marginal maximum likelihood estimation to condition on the latent A factor, rather than actually estimating genetic factor scores. This latent variable approach avoids the loss of information incurred by a two-step analysis of deriving and analyzing factor scores, when the scores may not be identically distributed.

In this article, we turn to consider the SEM implementations of genotype-environment interaction, where the effects of latent variables (genotypes and environments) are moderated by measured genotypes at the individual level. With high throughput genotyping technologies, it is feasible to measure genetic variants in large samples. These usually are single nucleotide polymorphisms (SNPs), a single nucleotide in the genome sequence that varies between people. SNPs that are associated with a phenotype of interest can be combined into polygenic scores (PGS) which, as an observed variable, provides a (partial) handle on the otherwise latent genetic variable underlying a phenotype of interest (Winkler et al. 2014; Tam et al. 2019; Choi et al. 2020).

Here we consider a model that incorporates PGS in structural equation models for data collected in mono- and dizygotic twin pairs to test for genotype-environment interaction. In the approach we take in this paper a PGS, an observed genetic variable, functions as the moderator. This allows us to test the hypothesis that the effect of the environment on the phenotype of interest depends on an individual’s genotype. Because we apply the model in the context of twin data, we test both A × C and A × E interaction. The PGS may reflect the genetic contribution to the phenotype that is analyzed, e.g., a PGS for major depression disorder (MDD) based on a genome-wide association study (GWAS) for MDD, or may reflect the genetic contribution to a different phenotype that is relevant to the phenotype of interest. Most human traits are polygenic and multiple traits tend to be correlated for genetic reasons (Brainstorm Consortium et al. 2018). Therefore, PGSs for one trait tend to correlate with a variety of other traits.

This paper is organized as follows. First, we introduce classical twin design and the concept of PGSs. We refrain from a detailed description of PGS estimation, as excellent resources are available (Choi et al. 2020). We then describe a test of genotype-environment interaction in which the PGS functions as a genetic moderator. We present a simulation study, in which we address parameter recovery and statistical power of the Environment-by-PGS interaction model, and end with an empirical study in which we apply this model to data on childhood anxiety and negative affect.

### The twin classical design

**Figure 1.**
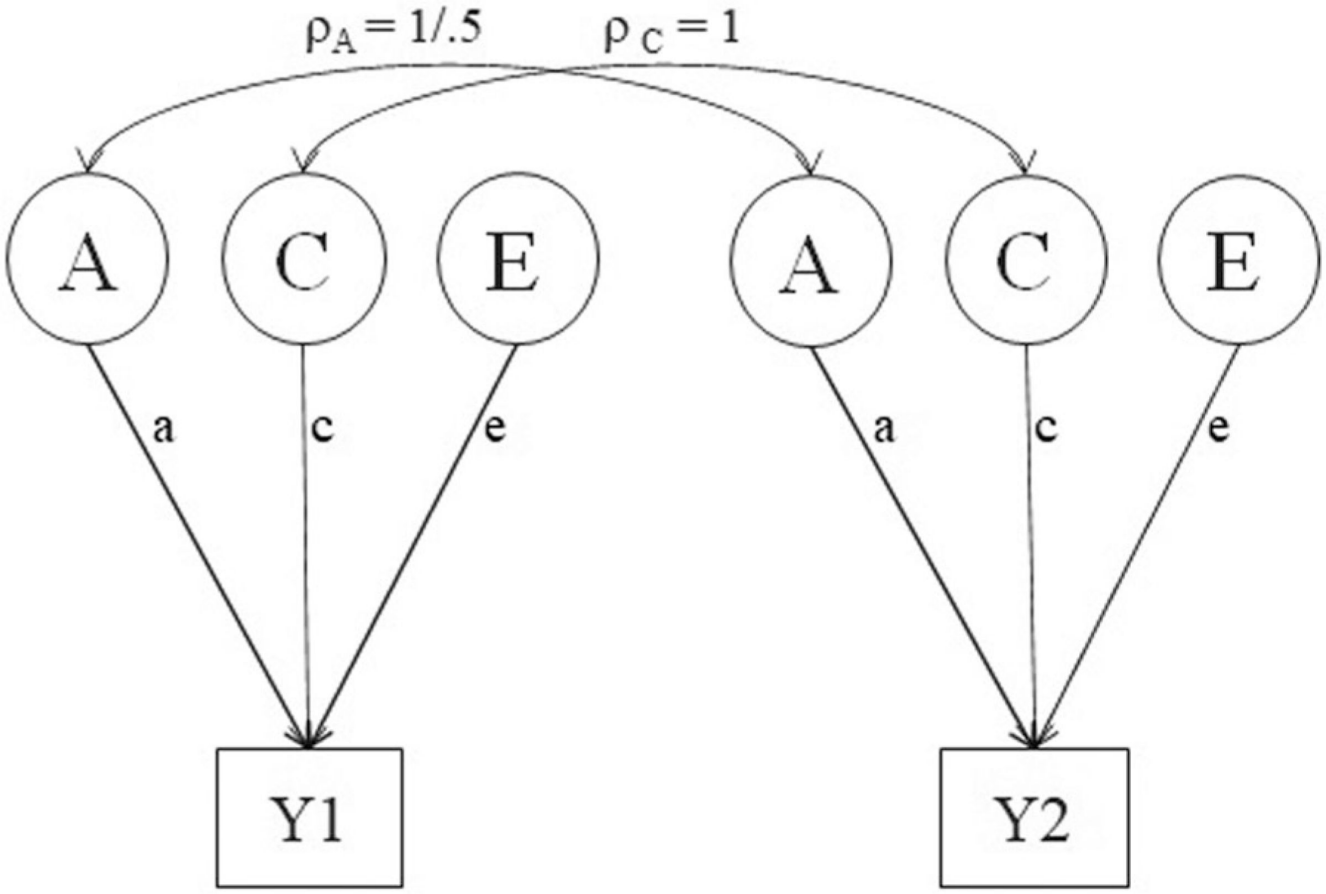
Path diagram of the ACE twin model. Y indicates the measured phenotype (Y1 and Y2 are the phenotypes of twin 1 and twin 2). Squares denote observed variables; circles denote latent variables. $\rho_{\text{A}}$ denotes the degree of genetic similarity (correlation) between members of a twin pair (1 for MZ and 0.5 for DZ twins); A, C, and E are uncorrelated and represent the genetic, shared environmental, and unshared environmental factors; a, c, and e are the additive genetic, shared environmental, and unshared environmental path coefficients respectively. The variance of the latent variables is 1.

The classical twin design (CTD) is applied to decompose the phenotypic covariance matrix of twins into genetic and environmental covariance matrices (Bruins et al. 2022a). The basis for the decomposition is the difference in genetic relatedness between two types of twins: monozygotic (MZ) twins arise from a single fertilized egg and therefore share 100% of their DNA, except for rare somatic mutations that arise in one member of a twin pair, but not in the other. Dizygotic (DZ) twins share on average 50% of their alleles at segregating loci. Both types of twins may share environmental influences, including prenatal exposures, which may contribute to their phenotypic resemblance. All environmental influences that contribute to phenotypic differences within pairs are referred to as unshared (unique) environments. We typically distinguish four sources of variance, namely additive genetic (A) variance (${\text{a}}^{\text{2}}$), non-additive or dominance genetic (D) variance (${\text{d}}^{\text{2}}$), common environmental (C) variance (${\text{c}}^{\text{2}}$), and unshared (E) environmental variance (${\text{e}}^{\text{2}}$) that typically also includes measurement error variance. The model including all four variance components is not identified with only data from MZ and DZ twin pairs. Here, we proceed with an ACE model, where the phenotypic variance then equals $\sigma_{{\text{Y}}^{2}}={\text{a}}^{2}+{\text{c}}^{2}+{\text{e}}^{2}$, when assuming no covariance or interaction among variance components (Eaves 1977). Figure 1 displays a path diagram of the univariate ACE twin model. The expected MZ and DZ covariance matrices are:

(1) $$\Sigma_{\text{MZ}}^{2}=\left(\begin{array}{cc} \sigma_{\text{MZ}}^{2} & \sigma_{\text{MZ}1,\text{MZ}2}\\ \sigma_{\text{MZ}1,\text{MZ}2} & \sigma_{\text{MZ}}^{2} \end{array}\right)$$

$$=\left(\begin{array}{cc} {\text{a}}^{\text{2}}{+\text{c}}^{\text{2}}{+\text{e}}^{\text{2}} & {\text{a}}^{\text{2}}{+\text{c}}^{\text{2}}\\ {\text{a}}^{\text{2}}{+\text{c}}^{\text{2}} & {\text{a}}^{\text{2}}{+\text{c}}^{\text{2}}{+\text{e}}^{\text{2}} \end{array}\right)$$

(2) $$\Sigma_{\text{DZ}}^{2}=\left(\begin{array}{cc} \sigma_{\text{DZ}}^{2} & \sigma_{\text{DZ}1,\text{DZ}2}\\ \sigma_{\text{DZ}1,\text{DZ}2} & \sigma_{\text{DZ}}^{2} \end{array}\right)$$

$$\text{=}\left(\begin{array}{cc} {\text{a}}^{\text{2}}{+\text{c}}^{\text{2}}{+\text{e}}^{\text{2}} & \text{0}{\text{.5a}}^{\text{2}}{+\text{c}}^{\text{2}}\\ \text{0}{\text{.5a}}^{\text{2}}{+\text{c}}^{\text{2}} & {\text{a}}^{\text{2}}{+\text{c}}^{\text{2}}{+\text{e}}^{\text{2}} \end{array}\right)$$

The contributions of A to the phenotypic covariance between twins correspond to the expected proportions of shared alleles in the MZ (1) and DZ twin pairs (0.5).

### Observed genetic variables: Polygenic scores

At a locus with 2 alleles, denoted B and b, we can observe 3 genotypes: bb, bB or Bb, and BB, which are coded 0 (bb), 1 (Bb or bB), and 2 (BB). An individual’s PGS is calculated as the weighted linear combination of these values from all SNPs that contribute to a phenotype (Choi et al. 2020; Wray et al. 2021). The weights are based on effects estimated in genome-wide association studies (see, e.g., Vilhjálmsson et al. 2015). Suppose that K SNPs contribute to the genetic variance of a trait. We express an individual’s (i) phenotype (Y) as:

(3) $${\text{Y}}_{\text{i}}={\text{b}}_{0}+\;\sum_{\text{k}=1}^{\text{K}}{\text{b}}_{\text{k}}{\text{SNP}}_{\text{ki}}+{\text{cC}}_{\text{i}}+{\text{eE}}_{\text{i}},$$

where C and E are standardized latent variables, SNP (a measured locus with three distinct genotypes) is coded 0, 1, or 2, and ${\text{b}}_{\text{K}}$ is the regression coefficient of the kth SNP. The phenotypic variance is expressed as

(4) $$\sigma_{\text{Y}}^{2}=\sum_{\text{k}=1}^{\text{K}}{\text{b}}_{\text{k}}^{2}\sigma_{\text{SNPk}}^{2}+{\text{c}}^{2}+{\text{e}}^{2}$$

In practice, we will have a subset comprising P of the K SNPs, that are used in calculating the PGS, and with L = K-P unmeasured SNPs contributing to the phenotypic variance. Then

(5) $$\sigma_{\text{Y}}^{2}=\sum_{\text{p}=1}^{\text{P}}{\text{b}}_{\text{p}}^{2}\sigma_{\text{SNPp}}^{2}+\sum_{\text{l}=1}^{\text{L}}{\text{b}}_{1}^{2}\sigma_{\text{SNPl}}^{2}+{\text{c}}^{2}+{\text{e}}^{2}$$

$$={\text{a}}_{\text{p}}^{2}+{\text{a}}_{\text{L}}^{2}+{\text{c}}^{2}+{\text{e}}^{2}$$

where ${\text{a}}_{\text{P}}^{2}=\sum_{p=1}^{\text{P}}{\text{b}}_{\text{p}}^{2}\sigma_{\text{SNPp}}^{2}$ is the genetic variance of Y explained by the PGSs, and ${\text{a}}_{\text{L}}^{2}=\sum_{l=1}^{\text{L}}{\text{b}}_{1}^{2}\sigma_{\text{SNPl}}^{2}$ is the residual genetic variance of Y.

### Environment-by-PGS interaction

**Figure 2.**
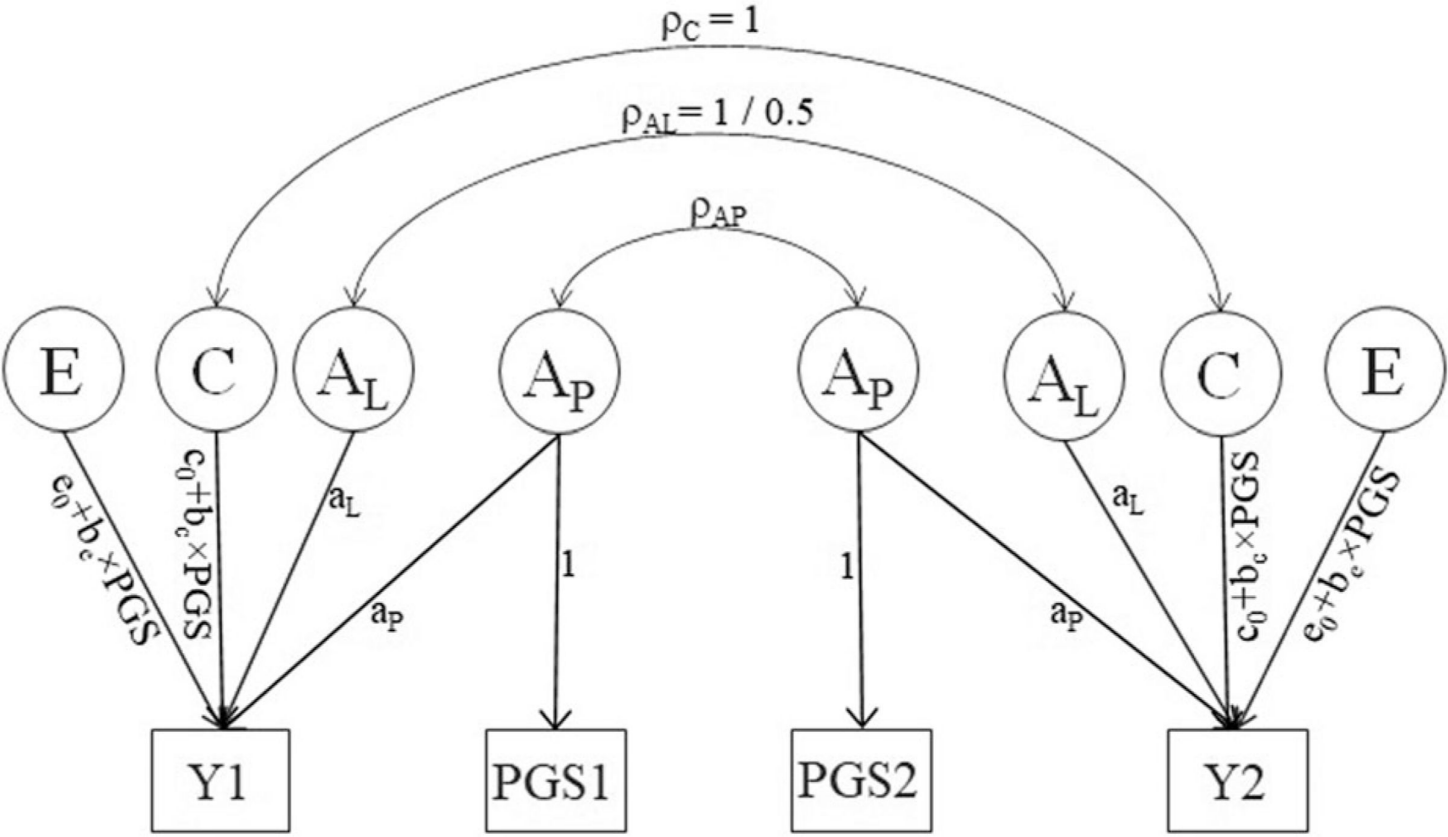
Path diagram for environment-by-PGS interaction model. Y denotes the phenotypes of twin 1 and twin 2. PGS1 and PGS2 are polygenic scores of twins 1 and 2. Squares denote observed variables and circles denote latent variables. ${\text{A}}_{\text{P}}$ and the PGS are identical (one-to-one relationship), and ${\text{A}}_{\text{L}}$, C, and E are the latent variables with effects ${\text{a}}_{\text{L}}$, ${\text{c}}_{0}$, and ${\text{e}}_{0}$, respectively. The parameter a_P_ denotes the main effect of the PGS on the phenotype; ${\text{b}}_{\text{c}}$ and ${\text{b}}_{\text{e}}$ are the interaction parameters. The variances of all latent variables are 1. The parameter $\rho_{\text{AL}}$ denotes the correlation between the additive genetic variables (1 for MZ twins and 0.5 for DZ twins), $\rho_{\text{AK}}$ is the correlation between the PGSs.

We propose to model the PGS × C and PGS × E interaction, as depicted in Figure 2. The moderation (or interaction) parameters, denoted ${\text{b}}_{\text{c}}$ and ${\text{b}}_{\text{e}}$, accommodate the dependency of the C and E effects on the PGS. We test this moderation by specifying the effects of C and E on the phenotype as a function of the PGS: $\text{C}={\text{c}}_{0}+{\text{b}}_{\text{c}}\times \text{PGS}$ and $\text{E}={\text{e}}_{0}+{\text{b}}_{\text{e}}\times \text{PGS}$. In the absence of environment-by-PGS interaction, the parameters ${\text{c}}_{0}$ and ${\text{e}}_{0}$ are equal to the parameters c and e in Figure 1. In Figure 2, the proportion of genetic variance captured by the PGS is ${\text{R}}_{\text{A}}^{2}={\text{a}}_{\text{p}}^{2}/\left({\text{a}}_{\text{L}}^{2}+{\text{a}}_{\text{p}}^{2}\right)$. The variance-covariance matrices are

(6) $$\Sigma_{\text{MZ}}=\left(\begin{array}{cc} \sigma_{\text{MZ}}^{2} & \sigma_{\text{MZ}1,\text{MZ}2}\\ \sigma_{\text{MZ}1,\text{MZ}2} & \sigma_{\text{MZ}}^{2} \end{array}\right)$$

$$\text{=}\left(\begin{array}{cc} {\text{a}}_{\text{L}}^{\text{2}}{+\text{a}}_{\text{P}}^{\text{2}}\text{+}{\left({\text{c}}_{\text{0}}{+\text{b}}_{\text{c}}\text{×PGS}\right)}^{\text{2}}\text{+}{\left({\text{e+b}}_{\text{e}}\text{×PGS}\right)}^{\text{2}} & {\text{a}}_{\text{L}}^{\text{2}}{+\text{a}}_{\text{P}}^{\text{2}}\text{+}{\left({\text{c}}_{\text{0}}{+\text{b}}_{\text{c}}\text{×PGS}\right)}^{\text{2}}\\ {\text{a}}_{\text{L}}^{\text{2}}{+\text{a}}_{\text{P}}^{\text{2}}\text{+}{\left({\text{c}}_{\text{0}}{+\text{b}}_{\text{c}}\text{× PGS}\right)}^{\text{2}} & {\text{a}}_{\text{L}}^{\text{2}}{+\text{a}}_{\text{P}}^{\text{2}}\text{+}{\left({\text{c}}_{\text{0}}{+\text{b}}_{\text{c}}\text{×PGS}\right)}^{\text{2}}\text{+}{\left({\text{e}}_{\text{0}}{+\text{b}}_{\text{e}}\text{×PGS}\right)}^{\text{2}} \end{array}\right)$$

(7) $$\Sigma_{\text{DZ}}=\left(\begin{array}{cc} \sigma_{\text{DZ}}^{2} & \sigma_{\text{DZ}1,\text{DZ}2}\\ \sigma_{\text{DZ1},\text{DZ}2} & \sigma_{\text{DZ}}^{2} \end{array}\right)$$

$$=\left(\begin{array}{cc} {\text{a}}_{\text{L}}^{2}+{\text{a}}_{\text{P}}^{2}+{\left({\text{c}}_{0}+{\text{b}}_{\text{c}}\times \text{PGS}\right)}^{2}+{\left({\text{e}}_{0}+{\text{b}}_{\text{e}}\times \text{PGS}\right)}^{2} & 0.5{\text{a}}_{\text{L}}^{2}+0.5{\text{a}}_{\text{P}}^{2}+{\left({\text{c}}_{0}+{\text{b}}_{\text{c}}\times \text{PGS}\right)}^{2}\\ 0.5{\text{a}}_{\text{L}}^{2}+0.5{\text{a}}_{\text{P}}^{2}+{\left({\text{c}}_{0}+{\text{b}}_{\text{c}}\times \text{PGS}\right)}^{2} & {\text{a}}_{\text{L}}^{2}+{\text{a}}_{\text{P}}^{2}+{\left({\text{c}}_{0}+{\text{b}}_{\text{c}}\times \text{PGS}\right)}^{2}+{\left({\text{e}}_{0}+{\text{b}}_{\text{e}}\times \text{PGS}\right)}^{2} \end{array}\right)$$

We investigate PGS × C and PGS × E interaction by testing $b_{\text{c}}=0$ and/or $b_{e}=0$. Because $R_{\text{A}}^{2}<1$, the estimated interaction parameters (${\text{b}}_{\text{c}}$, ${\text{b}}_{\text{e}}$) underestimate the true interaction parameter values. Considering A × C interaction, let A denote the total additive genetic variable (i.e., $A=A_{P}+A_{L}$, where $A_{P}$ is PGS), let $\beta_{\text{c}}$ denote the true interaction parameter value, and let bc denote the estimated interaction parameter value. The effect of C and its interaction with A on the genotype is $c_{0}+\beta_{\text{c}}\times A$, but we estimate ${\hat{c}}_{0}+b_{C}\times A_{P}={\hat{c}}_{0}+b_{C}\times \sqrt{R_{A}^{2}}\times \text{A}$. Thus, we underestimate the interaction parameter value by a factor of $\sqrt{R_{A}^{2}}$, such that ${\text{b}}_{\text{c}}=\beta_{\text{c}}\times \sqrt{R_{A}^{2}}=\beta_{\text{c}}\times R_{\text{A}}$, and we can find the true interaction parameter by dividing the estimate by $R_{A}$. The same applies to be and $\beta_{\text{e}}$:

(8) $$\beta_{\text{c}}=\frac{b_{c}}{R_{\text{A}}}\text{and}\;\beta_{e}=\frac{b_{e}}{R_{A}}$$

Note that this correction based on ${\text{R}}_{\text{A}}$ is only applicable if the PGS is based on a GWAS of the phenotype of interest. If the PGS is based on a phenotype different from the phenotype of interest, $R_{A}$ will be underestimated, and the resulting corrected interaction parameters will be inflated.

### Simulation study

We explored the power to detect genotype-environment interaction in a simulation study and assume the following: Random mating of parents of twins, for the trait that is analyzed MZ twins are not treated differently than DZ twins (equal environment assumption), no genotype-environment correlation, and the phenotypic data conditional on the genetic variable follow a multivariate normal distribution.

We considered various scenarios, in which we varied the genetic and environmental variance ($a^{2}$, $c_{0}^{2}$, $e_{0}^{2}$), the proportion of genetic variance explained by the PGSs $\left({\text{R}}_{\text{A}}^{2}\right)$, and the sample composition (MZ:DZ twin ratio). The interaction effect size (${\text{δ}}_{c}$, ${\text{δ}}_{e}$) indicates the change in the environmental variance (${\text{c}}^{\text{2}}$, ${\text{e}}^{\text{2}}$) for each standard deviation increase in the PGS, i.e., the phenotypic variance that is explained by the interaction parameter (${\text{δ}}_{\text{c}}=b_{\text{c}}^{2}+2b_{\text{c}}c_{0}$ and ${\text{δ}}_{e}=b_{e}^{2}+2b_{\text{e}}e_{0}$). The effect size was ${\text{δ}}_{c}={\text{δ}}_{e}=0.10$ over all simulations, and we simulated data for 4000 twin pairs. The power to detect environment-by-PGS interaction was calculated as the power to reject the model in which the non-zero interaction parameters ($b_{c}$, $b_{e}$) were fixed to 0, over 1,000 replications, given alpha = 0.05, for separate (1 df) tests and an omnibus (2 df) test of the interaction parameters. An overview of simulation parameters is presented in Table 2. The choice of the parameter values is based in part on empirical parameter values from an earlier study where we found that IQ moderated genetic and environmental influences on psychopathology in children (Bruins et al. 2022b).

To determine whether the power to detect an interaction effect depended on sample composition (i.e., the MZ: DZ ratio), we considered the following scenarios: an equal number of MZ and DZ pairs (MZ: DZ = 1: 1); the ratio in the population with more DZ than MZ pairs (MZ:DZ=1/3:2/3); and an overrepresentation of MZ pairs relative to DZ pairs, as often found in adult twin studies (MZ:DZ=2/3:1/3).

In addition, we determined the false positive rate by simulating data without an interaction effect and calculated the proportion of simulations in which the test of the interaction parameters had a p-value smaller than .05. To explore violation of the conditional multivariate distribution, we determined the false-positive rate on left-censored data. Specifically, we introduced a floor effect by assigning a score corresponding to the quantile associated with the probability of 0.15 to the lowest 15% of the phenotypic data, and again calculated the proportion of false positive results. We considered left-censoring because many measures of psychopathology display floor effects in population-representative samples.

All analyses were conducted in R 3.6.0 (R Core Team 2018) in the R-packages doSNOW (Analytics and Weston 2014), foreach (Calaway et al. 2015), MASS (Venables and Ripley 2013) for the simulation study, and OpenMx 2.17.1 for genetic model fitting (Neale et al. 2016). R scripts are available at Open Science Framework (OSF), *via* doi: 10.17605/OSF.IO/GB7WQ

## Results of the simulation study

**Figure 3.**
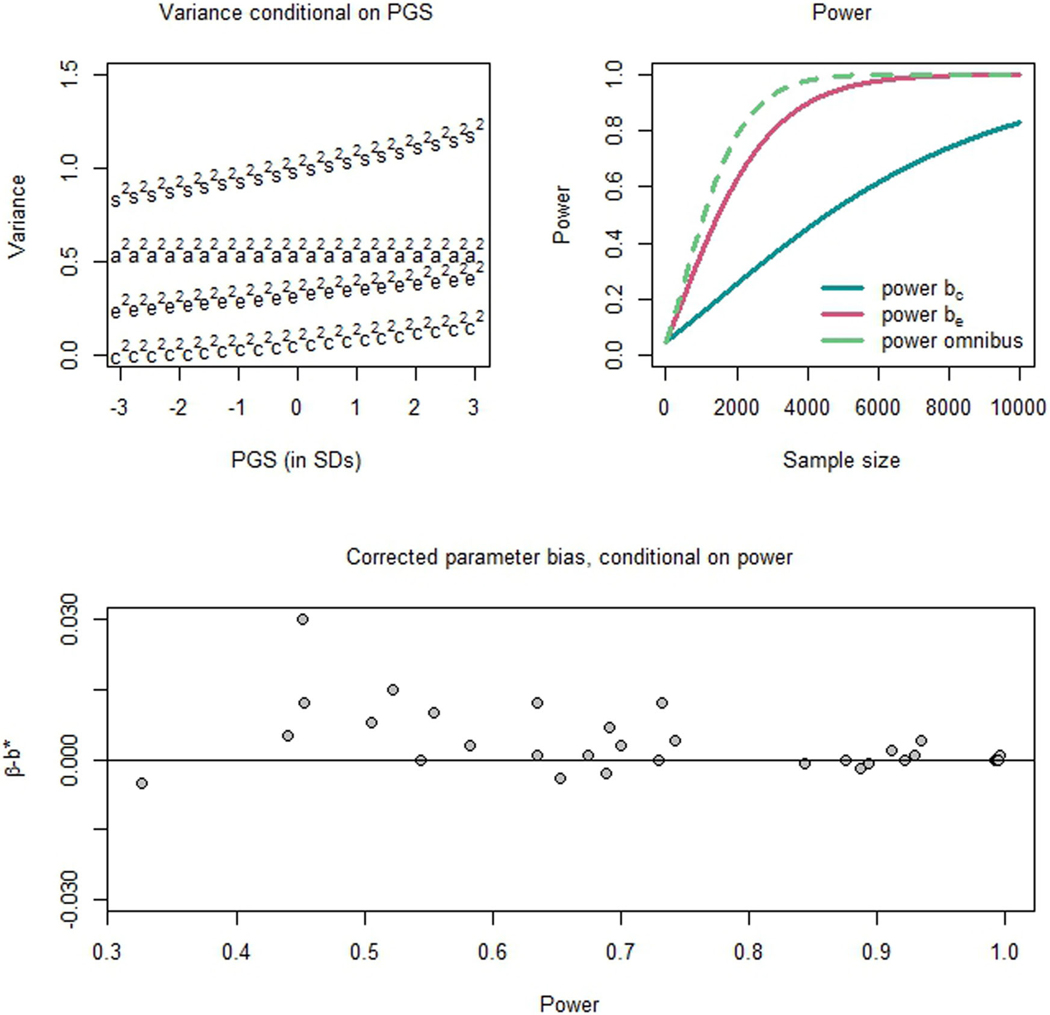
Results from the simulation study. **Top-left:** Genetic and environmental variance conditional on the PGS, from simulation 1. X-axis is the PGS (in SDs), y-axis is variance. ${\text{a}}^{\text{2}}$= total additive genetic variance $\left({\text{a}}_{\text{L}}^{2}+{\text{a}}_{\text{p}}^{2}\right)$, ${\text{c}}^{\text{2}}$= common environmental variance, ${\text{e}}^{\text{2}}$= unshared environmental variance, and ${\text{s}}^{2}$= total phenotypic variance. **Top-right:** The power to detect a non-zero ${\text{b}}_{\text{c}}$, ${\text{b}}_{\text{e}}$, and ${\text{b}}_{\text{omnibus}}$ parameter, in simulation 1. X-axis is number of twin pairs (MZ + DZ), y-axis is statistical power. **Bottom:** The discrepancy between the true interaction parameter (β as simulated) and the corrected estimate (i.e., $\text{b}/{\text{R}}_{\text{A}}$), conditional on the simulation power. Power is given on the X-axis, and the bias is shown on the y-axis $\left(\beta -b^{*}\right)$.

The power to detect the environment-by-PGS interaction effect depended on the A, C, and E variance, and increased with a greater proportion of genetic variance explained by the PGSs $\left({\text{R}}_{\text{A}}^{2}\right)$. We present the results of the simulation study in Table 3. In general, power to detect E-by-PGS interaction was greater than the power to detect C-by-PGS interaction, and the omnibus (2 df) test of ${\text{b}}_{\text{c}}$ and ${\text{b}}_{\text{e}}$ had greater power to detect environment-by-PGS interaction than the 1 df tests of individual interaction parameters (especially when the power for the individual tests was relatively modest, e.g., in simulations 2 and 8). Power also increases when the relative effect size increases. That is, the greater the ratio of parameters (i.e., ${\text{b}}_{\text{c}}/{\text{c}}_{0}$ and ${\text{b}}_{\text{e}}/{\text{e}}_{0}$) the greater the power to detect an environment-by-PGS interaction effect. Power was slightly lower when the MZ:DZ ratio was unbalanced (i.e., other than 1:1; see Supplementary Table 1). Figure 3 displays examples of conditional variance and power (based on simulation 1), and parameter bias in the interaction parameters (stemming from ${\text{R}}_{{\text{A}}^{\text{2}}}$<1).

Given multivariate normal phenotypic data (conditional on the PGS), the average false-positive rate is approximately equal to the alpha of 0.05 (fluctuating from 0.03 to 0.07 across simulations). However, when we introduced a floor effect in the data by collapsing the lowest 15% of the phenotypic scores into a single minimum score, the false positive rate ranged from 0.5 to 1 across simulations (see Supplementary Table 2).

### Empirical application to childhood anxiety and negative affect

We analyzed data on anxiety and affective problems from seven-year-old twins. These are heritable traits (Middeldorp et al. 2005; Wesseldijk et al. 2017). Bruins et al. (2022a, 2022b) found that genetic and/or environmental effects on anxiety and affect at age 7 were moderated by a total IQ score, as assessed by psychometric intelligence tests for children. Here, we tested whether environmental effects on anxiety and effect are moderated by a PGS of IQ. The discovery GWA study (Savage et al. 2018) published the effect sizes for SNPs for IQ in adults and children, which we used to calculate PGSs for the twins in our sample. The second series of tests is considered a PGS based on the currently largest discovery GWAS for Major Depressive Disorder (Wray et al. 2018). These discovery GWASs were mainly carried out in adult participants, but there is substantial genetic stability from early childhood to adulthood for IQ as well as for symptoms of anxiety and depression (e.g., Nivard et al. 2015), indicating that largely the same genetic variants are expressed in children and adults.

### Participants

Genotype and phenotype data were available for 1391 MZ and 1185 DZ twin pairs who participate in the Young Netherlands Twin Register (YNTR). Participants of the YNTR are recruited at birth, and their parents complete surveys on their development until the twins reach the age of 12 years. In this study, we analyzed data on anxiety and affective problems, as reported by mothers on their 7-year-old children (Van Beijsterveldt et al. 2013).

### Measurements

Anxiety and negative affect were assessed by the DSM-oriented scales of the Child Behavior Check List (CBCL; Achenbach et al. 2003). The CBCL includes 112 items concerning specific problem behaviors that are rated on a scale from 0—2, where 0 = not true; 1 = somewhat or sometimes true; and 2 = very true or often true. The affect scale (“affective problems”) consisted of 13 items, and the anxiety scale (“anxiety problems”) consisted of 6 items. Both scales have good test-retest reliability over 8 days (*r*= 0.84 for affective problems and *r*= 0.80 for anxiety problems), and acceptable to good internal consistency (α = 0.82 for affective problems and α = 0.72 for anxiety problems; Achenbach 2001). Total scale scores were constructed with item response theory, using a graded response model (Samejima 1997). The distribution of the scores is characterized by left censoring (a floor effect), caused by overuse of the “not true” category. We return to the effect of this censoring on the results of our analyses below.

The PGSs were based on the discovery GWA meta-analysis of (Savage et al. 2018) for intelligence, and the GWAS of (Wray et al. 2018) for major depressive disorder. We detail our procedure for PGS construction in the supplementary methods. We scaled the PGSs to have a mean of 0 and a variance of 1, and we reverse-coded the IQ PGS such that it correlated positively with negative affect and anxiety.

The best-fitting twin model for anxiety includes the influence of A and E, while for negative affect we also find a contribution of C (Bruins et al. 2022b). Therefore, we tested only E-by-PGS interaction for anxiety, and both E-by-PGS and C-by-PGS interaction for negative affect. As demonstrated in the simulation results above, censoring can lead to false positive interaction parameters. When we detected environment-by-PGS interaction, we took the effect of censoring into account by re-analyzing the data while explicitly modeling the left-censored phenotypic distribution in OpenMx (see for example, de Zeeuw et al. 2019). For all analyses, we used a significance threshold of α = 0.05.

## Results application to empirical data

The PGSs for IQ and MDD each captured 0.3% of the additive genetic variance of anxiety, and 0.4% of the additive genetic variance of negative affect. The correlation between these PGSs equaled 0.135. Results for negative affect suggested some interesting interaction effects, both for the IQ-PGS and the MDD-PGS. There was C-by-PGS interaction, where shared environmental variance for negative affect changed with a higher PGS for negative affect or lower IQ, such that most shared environmental variance of negative affect can be observed in the tails of the PGS distribution. For negative affect, there was also evidence for E-by-PGS interaction, where unshared environmental variance increased with a lower PGS for IQ. The E-by-PGS interaction coefficient for the MDD PGS was not significant (*p* = 0.063). For anxiety, we observed no environment-by-PGS interaction.

**Figure 4.**
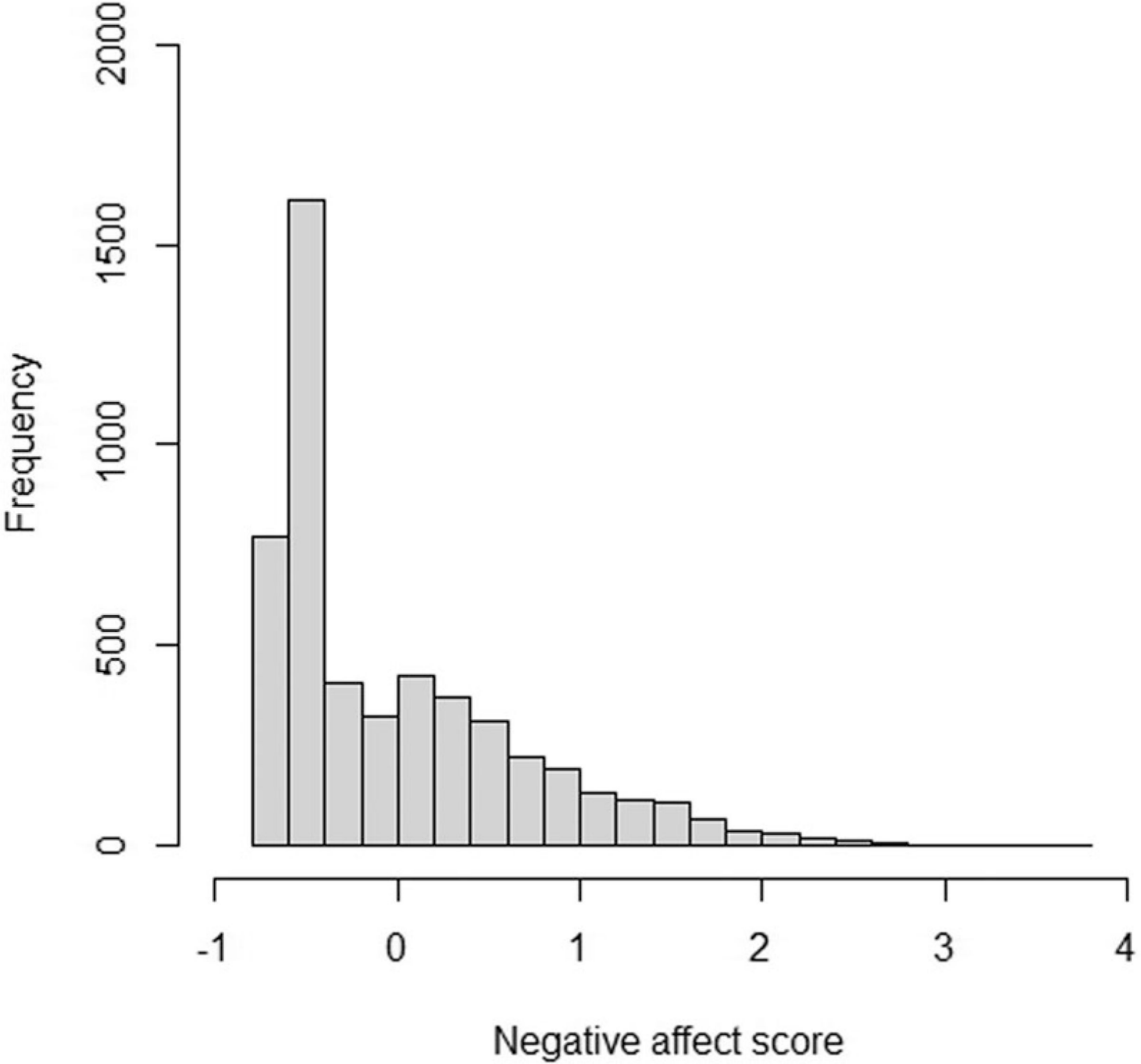
Histogram of negative affect scores.

**Figure 5.**
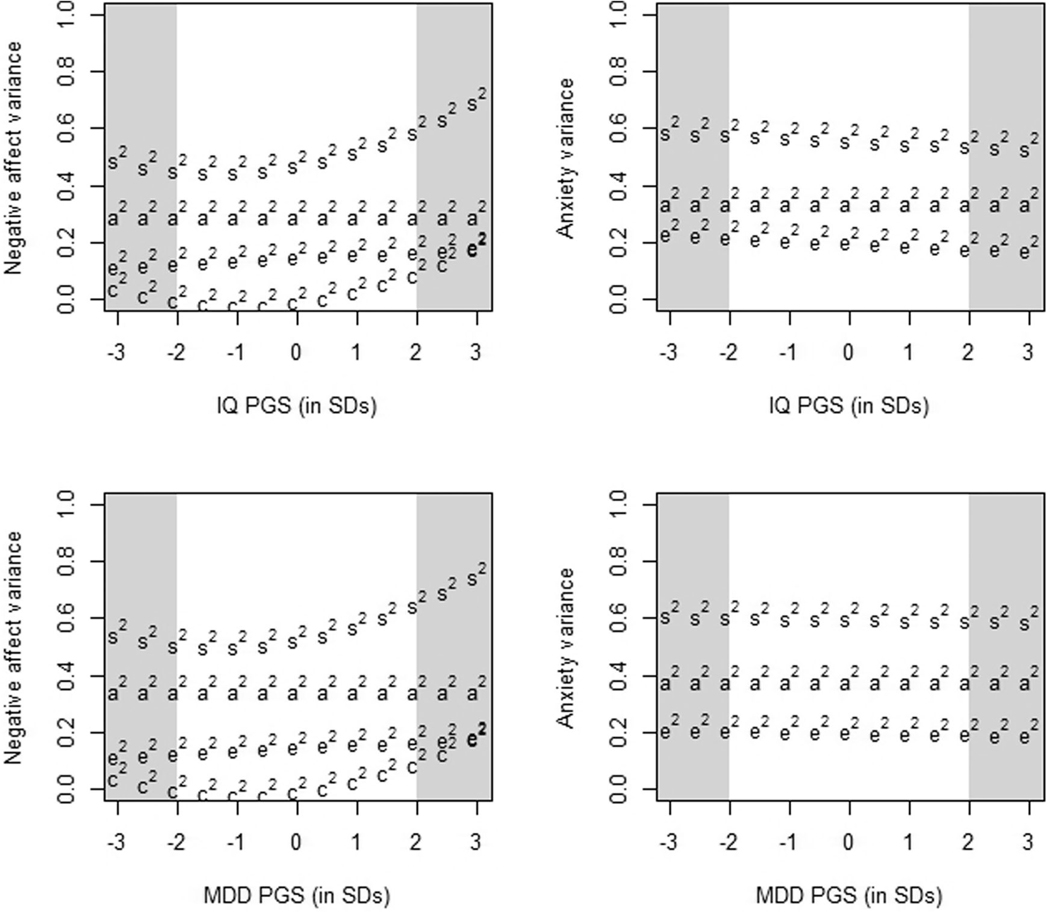
Genetic, environmental, and total variance of negative affect (left panes) and anxiety (right panes), conditional on the PGSs of IQ (top panes) and MDD (bottom panes). ${\text{a}}^{2}$= total additive genetic variance $\left({\text{a}}_{\text{L}}^{2}+{\text{a}}_{\text{p}}^{\text{2}}\right)$, ${\text{c}}^{\text{2}}$= shared environmental variance, ${\text{e}}^{\text{2}}$= unshared environmental variance, and s^2^ = total phenotypic variance. The variance components are based on the parameter estimates from the analysis without the censoring correction. The shaded blocks indicate a PGS ±2–3 SD’s from the mean.

In the simulation study, we demonstrated that the false-positive rate drastically increases when there is a floor effect in the data. In our empirical data, 15% of the subjects have the minimum score on negative affect (see Figure 4). To assess how this floor effect influences the results, we re-analyzed the negative affect data, while explicitly modeling the left-censored phenotypic distribution. We still found that there was C-by-IQ-PGS interaction after correcting for censoring, but C-by-MDD-PGS interaction and E-by-PGS interaction disappeared. The model fitting results are in Table 4. The conditional variance components are shown in Figure 5.

Given that the PGSs explained less than 1% of the genetic variance of anxiety and negative affect, and that the sample size was small compared to the simulated sample size, the power to detect a small effect was low, and the obtained estimates are rather imprecise. When we correct the interaction parameters for the proportion of genetic variance explained by the PGSs, the resulting corrected estimates are implausibly large. This is in line with our simulations, in which underpowered studies yield imprecise estimates. When corrected for ${\text{R}}_{\text{A}}$, these corrected estimates may not reflect the true effect.

## Discussion

Our aim was to present the environment-by-PGS interaction model and to discuss its implementation in combination with the classical twin design. We looked at the power to detect environment-by-PGS interaction for two different types of environmental influences, those that create a resemblance between children from the same household (common environment) and those that are unique to a person (unique environment). We found that the detection of genotype-environment interaction depends on the size of the A, C, and E effects, and on the genetic variance explained by PGSs. We found that the power to detect E-by-PGS generally was greater than the power to detect C-by-PGS interaction, and a 2 df omnibus test of both C- and E-by-PGS interaction had the greatest statistical power.

The finding that the power to detect E-by-PGS interaction is generally greater than the power to detect C-by-PGS interaction is in line with previous results of Molenaar et al (D. Molenaar et al. 2012; D. Molenaar and Dolan 2014). When there is no particular interest in the type of interaction (E-by-PGS or C-by-PGS), we recommend testing for environment-by-PGS interaction with a 2 df omnibus test. The power to detect environment-by-PGS interaction also depends on the proportion of additive genetic variance (${\text{R}}_{{\text{A}}^{\text{2}}}$) that a PGS explains. As of current, this proportion is typically small, but with ongoing progress in GWAS meta-analyses, the ${\text{R}}_{{\text{A}}^{\text{2}}}$ is likely to increase. We noted that the estimates of the interaction parameters (${\text{b}}_{\text{c}}$ and b_e_) depend on the genetic variance explained by the PGS (${\text{R}}_{{\text{A}}^{\text{2}}}$). However, as we demonstrated, it is simple to correct these parameter estimates, given that the parameters are estimated with reasonable accuracy.

We applied the environment-by-PGS interaction model to empirical data on childhood anxiety and negative affect, recognizing that although the study included 5,152 children with genotype and phenotype data, the power calculations from our simulations and the highly skewed phenotypic data suggest that caution is required. We considered two PGSs. Based on earlier work in which phenotypic IQ was tested as a moderator, we considered an IQ PGS, and the other PGS was based on a large GWAS in adults for major depressive disorder. Although these two PGSs explained less than 1% of the genetic variance, we detected C-by-PGS interaction for negative affect, where a PGS for lower IQ was associated with more shared environmental variance contributing to individual differences in negative affect. We accommodated the floor effect in the phenotypic data by modeling a censored distribution. However, our data did not follow a censored normal distribution, and our empirical results may yet be influenced by distributional issues.

Most genotype-environment interaction studies in behavioral science have focused on how genetic effects are expressed differentially depending on the environment (environmental moderation of genetic effects; Hagenbeek et al. 2022; Willoughby et al. 2023). The environment-by-PGS interaction method provides an explicit test of the hypothesis that individuals may have a differential sensitivity to environmental circumstances depending on their genotype. One advantage of this method is that a prior hypothesis concerning which specific environments have differential effects across levels of genetic sensitivity is not required. As such, studies using this method can serve as a first step in determining whether environmental effects differ over levels of genetic sensitivity, and if so, follow-up studies can focus on identifying specific environmental circumstances.

The present method is a contribution to the growing body of methods that exploit measured genetic information. While these strategies have gained popularity for the study of genotype-environment correlation (Dolan et al. 2021), its application in the study of genotype-environment interaction has been more limited (Hagenbeek et al. 2022). The incorporation of PGSs in the modeling of family data has already allowed for the development of new and more powerful tests of genotype-environment correlation. PGSs in family members have been leveraged to estimate the covariance between shared environment and genotype in twins and full sibs (Selzam et al. 2019; Balbona et al. 2021; Dolan et al. 2021) and in parents and offspring. (Kong et al. 2018; Bates et al. 2018; de Zeeuw et al. 2019).

Rathouz et al (2008) have presented a careful analysis of Purcell’s (2002) original bivariate moderation model. They demonstrated that a non-linear relationship between the moderator M and the phenotype may give rise to spurious A-by-M interaction. This spurious A-by-M interaction specifically concerns the moderation of the common A effects on the phenotype. By common A we mean the A component that is common to the moderator and the phenotype in the standard Cholesky representation of the Purcell model. However, in the present model, the effects of the PGS are not subject to moderation, and interaction is limited to phenotype-specific C and E effects. Hence, these concerns do not apply to the environment-by-PGS interaction model.

The tests of environment-by-PGS interaction, like most tests of interaction, are scale-dependent (Wagenmakers et al. 2012). That is, genotype-environment interaction estimates depend on the scaling of the phenotype, and positive results may be a function of “bad” scaling (inconsistent measurement precision across the scale) rather than true interaction (Eaves 2006). We considered a censored model, to accommodate the evident floor effects. While this is relatively simple to implement, we acknowledge there are other possibly more appropriate methods that one may consider to reduce the probability of false positive interaction tests. A potential solution to overcome scaling issues is to introduce a psychometric model to link phenotypic indicators (e.g., items) to the phenotypic latent variable. The test of interaction can then be conducted at the level of the latent variable (van den Berg et al. 2007; Molenaar and Dolan 2014). An advantage of the present approach to analyzing genotype-environment interaction based on measured PGSs is that it was relatively straightforward to incorporate in psychometric modeling.

A further possibility to improve the modeling of interaction effects on poorly-scaled measurements would be to use the standard errors of factor scores to moderate the residual variance of the trait. This approach has shown advantages in the context of factor analysis and seems appropriate to address scaling issues in studies of G × E interaction. Simulations are underway to explore this conjecture.

## Supplementary Material

Supplementary Methods

Supplementary Table 2

Supplementary Table 1

## Figures and Tables

**Table 1.** Glossary of terminology and notation.

Glossary	Notation

**Genotype-environment correlation.** Correlation between genes and environment that arises when exposure to certain environments is partially influenced by an individual’s genotype. **Genotype-environment interaction.** Interaction between genotype and environment that can occur when the effects of the environment on a phenotype depend on genotype, or when the effects of the genotype on a phenotype depend on environment. **Single nucleotide polymorphism (SNP).** A common type of genetic variation that occurs when a single nucleotide (Adenine, Thymine, Cytosine, or Guanine) in the DNA sequence is altered. **Genome-wide association study (GWAS).** A study in which the associations between many genetic variants (often SNPs) and a phenotype are estimated. **Polygenic score (PGS).** Weighted linear combination of the SNPs that affect a phenotype. Also known as Polygenic Risk Score (PRS) or Polygenic Index (PGI). **Additive genetic effects.** Contribution of individual genes or alleles to a phenotype in a linear and additive manner. **Dominance genetic effects.** Non-additive genetic effects stemming from intra-locus interactions. **Common environmental effects.** Environmental effects that are shared between members of a twin pair. **Unique environmental effects.** Environmental effects that are not shared between members of a twin pair, typically also includes measurement error.	Y. Observed phenotypic variable. PGS. Measured PGS variable. $A_{L}$. Latent additive genetic variable. $a_{L}$. Additive genetic path coefficient. $A_{P}$. Latent variable representing the PGS. $a_{P}$. PGS path coefficient, denoting the effect of the PGS on phenotype Y. C. Common environmental variable. $c_{0}$. Common environmental path coefficient. $b_{c}$. C-by-PGS coefficient, denoting the interaction between the PGS and the common environment. E. Unique environmental variable. $e_{0}$. Unique environmental path coefficient. $b_{e}$. E-by-PGS coefficient, denoting the interaction between the PGS and the unique environment. $\rho_{AL}$. Additive genetic correlation between members of a twin pair. This corresponds to the proportion of shared genetic material (i.e., 1 for MZ twins and .50 for DZ twins). $\rho_{AP}$. Correlation between PGSs of a twin pair. Its value is expected to be 1 in MZ twins and .50, on average, in DZ twins. ${\text{δ}}_{c}$. Effect size of C-by-PGS interaction. Indicates the change in C variance with every standard deviation increase in the PGS. ${\text{δ}}_{e}$. Effect size of E-by-PGS interaction. Indicates the change in E variance with every standard deviation increase in the PGS. When the phenotype is scaled to have unit variance, ${\text{δ}}_{c}$ and ${\text{δ}}_{\text{e}}$ correspond to the proportion of phenotypic variance that is explained by the C- or E-by-PGS interaction. ${\text{R}}_{{\text{A}}^{\text{2}}}$. Proportion of additive genetic variance captured by the PGS.

*Note*. Parameters in italics are freely estimated in the environment-by-PGS interaction model.

**Table 2.** Parameter values in the simulation study.

Parameters settings			
Parameter	Values		

${\text{a}}^{2}$	.35	.55	
${\text{c}}_{0^{2}}$	.10	.35	
${\text{e}}_{0^{2}}$	.35	.50	
${\text{R}}_{{\text{A}}^{\text{2}}}$	.10	.20	
**Parameters in the power simulations**			
${\text{δ}}_{c}$	.10		
${\text{δ}}_{e}$	.10		
MZ/DZ ratio	2/3:1/3	1/3:2/3	1 : 1
**Parameters in the false positive rate simulations**			
${\text{δ}}_{c}$	0		
${\text{δ}}_{e}$	0		
MZ/DZ ratio	1 : 1		
Proportion of floor values	0	.15	

${\text{a}}^{\text{2}}$=total genetic variance; c02=shared environmental variance; e02=unshared environmental variance; RA2=proportion of genetic variance explained by PGS; ${\text{δ}}_{c}$=C-by-PGS interaction effect size; ${\text{δ}}_{e}$=E-by-PGS interaction effect size. The total number of MZ+DZ twin pairs was 4000.

**Table 3.** Results of simulation study, averaged over 1000 replications.

Parameter settings							Estimates				Relative effect size		Power			n .80		
	${\text{b}}_{\text{c}}$	${\text{b}}_{\text{e}}$	${\text{b}}_{{\text{c}}^{*}}$	${\text{b}}_{{\text{e}}^{*}}$	${\text{b}}_{\text{c}}/{\text{c}}_{0}$	${\text{b}}_{\text{e}}/{\text{e}}_{0}$	${\text{b}}_{\text{c}}$	${\text{b}}_{\text{e}}$	om	${\text{R}}_{{\text{A}}^{\text{2}}}$	${\text{a}}^{2}$	${\text{c}}_{0^{2}}$	${\text{e}}_{0^{2}}$	$\beta_{\text{c}}$	$\beta_{\text{e}}$	${\text{b}}_{\text{c}}$	${\text{b}}_{\text{e}}$	om

1	.1	.55	.10	.35	.131	.079	.044	.025	.143	.082	.14	.04	.45	.90	.98	12982	3300	2306
2	.1	.55	.10	.50	.131	.067	.050	.021	.161	.068	.16	.03	.45	.68	.92	13025	6433	3341
3	.1	.55	.35	.35	.079	.079	.030	.025	.094	.079	.05	.04	.52	.88	.97	10121	3565	2450
4	.1	.55	.35	.50	.079	.067	.023	.022	.074	.07	.04	.03	.33	.70	.86	24257	5994	4196
5	.1	.35	.10	.35	.131	.079	.042	.025	.134	.081	.13	.04	.58	.91	.99	8379	3119	1750
6	.1	.35	.10	.50	.131	.067	.043	.021	.139	.067	.14	.03	.51	.73	.97	10694	5513	2537
7	.1	.35	.35	.35	.079	.079	.028	.025	.089	.078	.05	.04	.55	.89	.99	9137	3331	2132
8	.1	.35	.35	.50	.079	.067	.027	.020	.084	.063	.05	.03	.44	.65	.90	13651	6832	3532
9	.2	.55	.10	.35	.131	.079	.063	.035	.143	.079	.20	.06	.73	.99	1	5489	1716	1084
10	.2	.55	.10	.50	.131	.067	.063	.031	.143	.071	.20	.04	.64	.94	1	7191	2796	1522
11	.2	.55	.35	.35	.079	.079	.036	.035	.080	.079	.06	.06	.64	.99	1	7183	1660	1252
12	.2	.55	.35	.50	.079	.067	.035	.029	.079	.065	.06	.04	.54	.89	.99	9420	3421	2110
13	.2	.35	.10	.35	.131	.079	.058	.036	.130	.080	.18	.06	.84	.99	1	3974	1508	831
14	.2	.35	.10	.50	.131	.067	.056	.030	.128	.068	.18	.04	.69	.93	1	6197	2879	1333
15	.2	.35	.35	.35	.079	.079	.037	.035	.083	.079	.06	.06	.74	.99	1	5333	1610	1074
16	.2	.35	.35	.50	.079	.067	.038	.030	.086	.067	.06	.04	.69	.92	1	6149	2994	1584

*Note*. nMZ = nDZ = 2000 (i.e., 4000 twin pairs, 8000 individuals). ${\text{R}}_{{\text{A}}^{\text{2}}}$ is the proportion of genetic variance explained by the PGSs. $\beta_{\text{c}}$ and $\beta_{\text{e}}$ are the true interaction parameters, ${\text{b}}_{\text{c}*}$, and ${\text{b}}_{\text{e}*}$ are the estimated interaction parameters, and ${\text{b}}_{\text{c}}$ and ${\text{b}}_{\text{e}}$ are the estimated parameters corrected for $R_{A}$, om stands for omnibus (2 df) test, n 0.80 is the number of twin pairs (MZ + DZ) required to detect an interaction effect with a power of 0.80 (given α= 0.05).

**Table 4.** Model fitting results of environment-by-PGS analyses for anxiety and negative affect.

	Phenotype	Test	−2LL	df	$\chi^{2}$	Δdf	p	AIC	b(b*)

IQ PGS	Anxiety		21236.79	8906				3424.79	
	Neg. affect	${\text{b}}_{\text{e}}$	21238.74 20332.49	8907 8904	1.94	1	.164	3424.74 2524.49	−0.01
		${\text{b}}_{\text{c}}$	20337.03	8905	4.54	1	.033	2527.03	.11 (1.92)
		${\text{b}}_{\text{e}}$	20336.36	8905	3.87	1	.049	2526.36	.01 (0.24)
	Neg. affect censored dist.		21158.46	8904				3350.46	
		${\text{b}}_{\text{c}}$	21163.61	8905	5.16	1	.023	3353.61	.13 (2.63)
		${\text{b}}_{\text{e}}$	21161.32	8905	2.87	1	.090	3351.32	.01
MDD PGS	Anxiety		21324.64	8906				3512.64	
	Neg. affect	${\text{b}}_{\text{e}}$	21324.87 20406.68	8907 8904	0.23	1	.631	3510.87 2598.68	−0.004
		${\text{b}}_{\text{c}}$	20413.73	8905	7.05	1	.008	2603.73	.11 (1.29)
		${\text{b}}_{\text{e}}$	20410.13	8905	3.45	1	.063	2600.13	.01
	Neg. affect censored dist.		21240.08	8904				3432.08	
		${\text{b}}_{\text{c}}$	21243.68	8905	3.60	1	.058	3433.68	.11
		${\text{b}}_{\text{e}}$	21241.44	8905	1.35	1	.245	3431.44	.01

-2LL is the −2 loglikelihood, df is degrees of freedom, b is the interaction parameter, and b* is the interaction parameter corrected by ${\text{R}}_{\text{A}}$. Neg. Affect censored dist. refers to the analyses in which we modeled a left-censored distribution.

## Data Availability

The Netherlands Twin Register data are stored in a repository and access to data may be provided for research purposes, after submitting a data request, which is evaluated by a data access committee (DAC). Detailed information about working with NTR data and the NTR repository can be found at https://ntr-data-request.psy.vu.nl/
