# Supplementary material for: Environment-by-PGS Interaction in the Classical Twin Design: An Application to Childhood Anxiety and Negative Affect
Source: Multivariate Behav Res. Author manuscript; Available in PMC 2024 Nov 22. (PMC11157501; doi:10.1080/00273171.2023.2228763)
Supplement: Supplementary Methods [file NIHMS1984787-supplement-Supplementary_Methods.docx]

**Supplementary methods for Environment-by-Polygenic Score Interaction in the classical twin design: an application to childhood anxiety and negative affect.**

The PGSs were based on the discovery GWA meta-analysis of (Savage et al. 2018) for intelligence, and the GWAS of (Wray et al. 2018) for major depressive disorder. We retained variants for which the effect allele frequency (EAF) was between 0.01 and 0.99. We aligned the GWA variants with the NTR reference for the 1000 genomes variants. Discovery variants that were not part of this reference were discarded. The processed summary statistics were taken as input for LDpred 0.9 (Vilhjálmsson et al. 2015). We estimated the target LD structure based on a subset of unrelated individuals in NTR and we selected a set of well-imputed variants in the NTR sample. The LD radius was set by dividing the number of variants in common by 12000. We specified N in the coordination step as median sample size. In the LDpred step, we specified the fractions P of causal variants with nonzero effect as 0.5, 0.3, 0.2, 0.1, 0.05, and 0.01, and an infinitesimal model, and calculated PGSs for all these thresholds using plink2 software (Chang et al. 2015). We selected the optimal value of P by performing a series of linear regression analyses of our phenotypes of interest (negative affect and anxiety) on the PGSs, and we retained the PGSs that explained the most variance in the negative affect and anxiety for further analysis. In our case, the PGSs based on the infinitesimal model and those with P values 0.1, 0.3 and 0.5 performed equally well. We chose to use the PGSs with P = 0.1 in our analyses.
