# Supplementary material for: Environment-by-PGS Interaction in the Classical Twin Design: An Application to Childhood Anxiety and Negative Affect
Source: Multivariate Behav Res. Author manuscript; Available in PMC 2024 Nov 22. (PMC11157501; doi:10.1080/00273171.2023.2228763)
Supplement: Supplementary Table 2 [file NIHMS1984787-supplement-Supplementary_Table_2.docx]

Supplementary Table 2. False positive rate with normally distributed data and left-censored data.

|  | **Parameter settings** | | | | **Normal distribution** | | | **Left-censored** | | |
| --- | --- | --- | --- | --- | --- | --- | --- | --- | --- | --- |
|  | **R_A_^2^** | **a_L_^2^** | **c_0_^2^** | **e_0_^2^** | **FPR bc** | **FPR be** | **FPR om** | **FPR bc** | **FPR be** | **FPR om** |
| 1 | 10 | 0.55 | 0.1 | 0.35 | 0.052 | 0.062 | 0.066 | 0.922 | 0.966 | 1 |
| 2 | 10 | 0.55 | 0.1 | 0.5 | 0.06 | 0.053 | 0.066 | 0.874 | 0.974 | 1 |
| 3 | 10 | 0.55 | 0.35 | 0.35 | 0.05 | 0.03 | 0.048 | 0.984 | 0.958 | 1 |
| 4 | 10 | 0.55 | 0.35 | 0.5 | 0.047 | 0.058 | 0.053 | 0.948 | 0.946 | 1 |
| 5 | 10 | 0.35 | 0.1 | 0.35 | 0.055 | 0.044 | 0.052 | 0.598 | 0.822 | 0.99 |
| 6 | 10 | 0.35 | 0.1 | 0.5 | 0.051 | 0.058 | 0.053 | 0.492 | 0.896 | 0.998 |
| 7 | 10 | 0.35 | 0.35 | 0.35 | 0.046 | 0.049 | 0.049 | 0.842 | 0.83 | 0.998 |
| 8 | 10 | 0.35 | 0.35 | 0.5 | 0.058 | 0.054 | 0.072 | 0.774 | 0.846 | 0.996 |
| 9 | 20 | 0.55 | 0.1 | 0.35 | 0.047 | 0.046 | 0.049 | 0.998 | 1 | 1 |
| 10 | 20 | 0.55 | 0.1 | 0.5 | 0.055 | 0.048 | 0.044 | 0.996 | 1 | 1 |
| 11 | 20 | 0.55 | 0.35 | 0.35 | 0.046 | 0.055 | 0.049 | 1 | 1 | 1 |
| 12 | 20 | 0.55 | 0.35 | 0.5 | 0.061 | 0.052 | 0.062 | 1 | 0.998 | 1 |
| 13 | 20 | 0.35 | 0.1 | 0.35 | 0.052 | 0.053 | 0.051 | 0.876 | 0.988 | 1 |
| 14 | 20 | 0.35 | 0.1 | 0.5 | 0.048 | 0.052 | 0.057 | 0.786 | 1 | 1 |
| 15 | 20 | 0.35 | 0.35 | 0.35 | 0.046 | 0.052 | 0.051 | 0.99 | 0.978 | 1 |
| 16 | 20 | 0.35 | 0.35 | 0.5 | 0.056 | 0.043 | 0.061 | 0.95 | 0.992 | 1 |

False positive rate is defined as the proportion of simulations in which a nonzero environment-by-PGS interaction coefficient was detected (bc, be, or both) while in truth, the coefficient was 0.
