# Supplementary material for: Environment-by-PGS Interaction in the Classical Twin Design: An Application to Childhood Anxiety and Negative Affect
Source: Multivariate Behav Res. Author manuscript; Available in PMC 2024 Nov 22. (PMC11157501; doi:10.1080/00273171.2023.2228763)
Supplement: Supplementary Table 1 [file NIHMS1984787-supplement-Supplementary_Table_1.docx]

Supplementary Table 1.

Power to detect environment-by-PGS interaction with MZ:DZ ratios of ⅔ : ⅓ and **=** ⅓ : ⅔

|  | **Parameter settings** | | | | **MZ:DZ =** ⅔ : ⅓ | | | **MZ:DZ =** ⅓ : ⅔ | | |
| --- | --- | --- | --- | --- | --- | --- | --- | --- | --- | --- |
|  | **R_A_^2^** | **a_L_^2^** | **c_0_^2^** | **e_0_^2^** | **pow bc** | **pow be** | **pow om** | **pow bc** | **pow be** | **pow om** |
| 1 | .10 | 0.55 | 0.1 | 0.35 | 0.46 | 0.93 | 0.98 | 0.46 | 0.82 | 0.97 |
| 2 | .10 | 0.55 | 0.1 | 0.5 | 0.40 | 0.74 | 0.91 | 0.37 | 0.63 | 0.89 |
| 3 | .10 | 0.55 | 0.35 | 0.35 | 0.38 | 0.93 | 0.97 | 0.41 | 0.82 | 0.94 |
| 4 | .10 | 0.55 | 0.35 | 0.5 | 0.35 | 0.75 | 0.88 | 0.35 | 0.62 | 0.84 |
| 5 | .10 | 0.35 | 0.1 | 0.35 | 0.59 | 0.94 | 1.00 | 0.59 | 0.85 | 0.99 |
| 6 | .10 | 0.35 | 0.1 | 0.5 | 0.50 | 0.76 | 0.96 | 0.49 | 0.65 | 0.96 |
| 7 | .10 | 0.35 | 0.35 | 0.35 | 0.47 | 0.93 | 0.98 | 0.49 | 0.86 | 0.98 |
| 8 | .10 | 0.35 | 0.35 | 0.5 | 0.42 | 0.75 | 0.92 | 0.42 | 0.65 | 0.91 |
| 9 | .20 | 0.55 | 0.1 | 0.35 | 0.72 | 1.00 | 1.00 | 0.72 | 0.98 | 1.00 |
| 10 | .20 | 0.55 | 0.1 | 0.5 | 0.62 | 0.95 | 1.00 | 0.62 | 0.88 | 1.00 |
| 11 | .20 | 0.55 | 0.35 | 0.35 | 0.60 | 1.00 | 1.00 | 0.61 | 0.98 | 1.00 |
| 12 | .20 | 0.55 | 0.35 | 0.5 | 0.52 | 0.95 | 0.99 | 0.53 | 0.87 | 0.99 |
| 13 | .20 | 0.35 | 0.1 | 0.35 | 0.85 | 1.00 | 1.00 | 0.85 | 0.99 | 1.00 |
| 14 | .20 | 0.35 | 0.1 | 0.5 | 0.73 | 0.96 | 1.00 | 0.74 | 0.91 | 1.00 |
| 15 | .20 | 0.35 | 0.35 | 0.35 | 0.73 | 1.00 | 1.00 | 0.74 | 0.99 | 1.00 |
| 16 | .20 | 0.35 | 0.35 | 0.5 | 0.63 | 0.95 | 1.00 | 0.62 | 0.90 | 1.00 |
